# Digital technologies and COVID-19: reconsidering lockdown exit strategies for Africa

**DOI:** 10.11604/pamj.2021.39.93.29773

**Published:** 2021-06-01

**Authors:** Itai Chitungo, Malizgani Mhango, Elliot Mbunge, Mathias Dzobo, Tafadzwa Dzinamarira

**Affiliations:** 1 Department of Laboratory Diagnostics and Investigative Science, Faculty of Medicine and Health Sciences, University of Zimbabwe, Harare, Zimbabwe,; 2 School of Public Health, University of Western Cape, 7535, Cape Town, South Africa,; 3 Department of Information Technology, Faculty of Accounting and Informatics, Durban University of Technology, P O Box 1334, Durban 4000, South Africa,; 4 ICAP at Columbia University, Kigali, Rwanda,; 5 School of Health Systems and Public Health, University of Pretoria, Pretoria, 0002, South Africa

**Keywords:** Africa, COVID-19, lockdown

## Abstract

Widespread vaccination provides a means for countries to lift strict COVID-19 restrictions previously imposed to contain the spread of the disease. However, to date, Africa has secured enough COVID-19 vaccine doses for less than 5% of its population. With widespread vaccination not on the horizon for Africa, there is a strong emphasis on non-pharmaceutical interventions which include movement restrictions (lockdowns). This general COVID-19 pandemic response of imposing lockdowns, however, neglects to factor in non-fatal consequences leading to disruption socio-economic wellbeing of the society at large. The economy in most African countries can no longer sustain lockdown restrictions. Some studies have indicated that a hard lockdown statistical value of the extra lives saved would be dwarfed by its long-term cost. At the same time not responding to the threat of the pandemic will cost lives and disrupts the social fabric. This paper proffers ways to mitigate the both and advocate for better policymaking that addresses specific challenges in defined communities thus yield higher population welfare.

## Perspectives

**Public health and economic impact of COVID-19 lockdowns:** despite the low number of reported cases in Africa [1,2], the effects of the coronavirus disease 2019 (COVID-19) lockdown measures on routine public health interventions have been well established on the African continent. For instance; disruption of malaria control efforts is estimated to double mortality with a concomitant greater damage in the preceding years [3]. In Rwanda, less than half of a surveyed population of people living with HIV/AIDS attended antiretroviral therapy (ART) medication collection clinic appointments during the first few months of a hard lockdown [4]. During the April to June 2020 hard lockdown in Zimbabwe, there was a 59% reduction in the number of clients tested for HIV and receiving their results, a 51% reduction in patients newly diagnosed with HIV initiated on ART, and a 29% decline in viral load sample collection [5].

The closure of borders and businesses has disrupted economies in African states with a regional average of about 5% in public revenue losses and total merchandise exports contracting by about 17% [6]. Further, inaccessible transport systems adversely affected supply chains, intra- and inter-regional African trade which had a ripple effect on productivity and coupled with decreased consumer spending [1]. On a household level, recent studies estimate that approximately 73 million Africans are acutely food insecure and this precarious situation has been amplified by the direct effects of the COVID-19 lockdown measures [7]. Severely affected are households that depend on the informal sector of the economy for survival. There is, therefore, an urgent need to devise COVID-19 control interventions that allow economic activities to continue whilst observing COVID-19 precautionary measures.

**Roll out of COVID-19 vaccination programs:** the roll-out of COVID-19 vaccine programs has brought a glimpse of hope, globally, as populations are expecting a shift in the trajectory of the pandemic and ease of lockdown restrictions as more people attain herd immunity. However, this glimmer of hope seems to be elusive to many in low-income countries particularly in Africa as they are far from realising the potential positive effect of a COVID-19 vaccine due to limited access and supply. As the vaccination campaigns roll out in other parts of the globe, Africa faces a mammoth task of ensuring the availability and accessibility of a safe, inclusive and sustainable distribution process that reaches frontline health workers, at-risk groups, and eventually all individuals to attain herd immunity [8]. It was expected that by 2021 approximately 12 billion doses of COVID-19 vaccines would be available globally. Despite the formation of COVID-19 Vaccines Global Access (COVAX), the future of Africa´s COVID-19 vaccination campaign remains unclear. For instance, COVAX has approximately 92 countries that are expecting to receive COVID-19 vaccines under this initiative. This means to reach the 20% WHO minimum target of immunisation coverage, there is a need for six to seven billion doses [9]. Simply put, this will be difficult if not impossible to achieve. Furthermore, the geographical location of the pharmaceutical companies that are manufacturing the COVID-19 vaccines puts Africa at a logistical disadvantage. It is important to note that more than 90% of all COVID-19 vaccines are manufactured in the USA, EU, India, and China whilst African countries are expected to import [10]. This exerts logistical stress and strain on the previously used models of supply chain especially with required cold chain conditions for some vaccines. Vaccine hesitancy and the emergency of new variants of the SARS-CoV-2 virus further slow down the attainment of herd immunity in Africa through vaccination [11].

**Africa, with the huge informal economy, what are the potential strategies to ease lockdown restrictions?** The impact of COVID-19 lockdown restrictions on the public health programs and economy underscores the need for urgent innovative strategies to ease restrictions while avoiding a resurgence of the disease. Widespread vaccination, a pathway exploited by richer countries as a means to ease restrictions through the attainment of herd immunity, is not on the horizon for Africa. In this perspective, we offer recommendations on how the continent can strategically ease the lockdowns that have slumped economic growth and disrupted other essential public health programs. Table 1 presents the adoption of digital technologies; implementation of smart lockdowns and COVID-19 vaccination passports as potential lockdown exit strategies for Africa.

**Table 1:** proposed lockdown exit strategies for Africa

Strategy	Concept
Smart lockdowns	Isolation of infection hotspots to break transmission cycle, while keeping the broader economy open
Digital contact tracing and surveillance	Utilising mobile data positioning to perform contact tracing and surveillance to enforce smart lockdowns.
Telehealth	To improve safety and continue medical provision in hotspots areas.
Digital education	For continuous education to pupils and students in hotspots areas.
Vaccine passports	To minimise imports of new infections from external sources while promoting broader economic relationship with other nations.

**a) Smart Lockdowns:** the adoption of lockdowns as a standard response to the COVID-19 pandemic has caused socio-economic turmoil on the continent. Scientists have proposed intermittent lockdowns commonly referred to as “smart lockdown” to lessen the impact of general restrictive measures [12]. The smart lockdown looks at disease hotspots and implements restrictive measures while economic activities continue in unaffected areas. Population in these hotspots are quarantined and monitored while movement into the area is limited to strictly essential goods and services. This differentiated approach to lockdowns breaks the transmission cycle allowing for broader economic activity to continue with minimal disruption and provide the population with a semblance of normalcy while waiting for the majority of the population to be vaccinated. However, this strategy requires more vigilance in the public sector as there is a need for constant monitoring to ensure these small outbreaks are contained and do not escalate to the point where harsher measures are needed [13]. The public sector in partnership with mobile network operators can increase surveillance vigilance and effectively implement smart lockdowns.

**b) Digital contact tracing and surveillance:** the success of smart lockdown hinges on the implementation of robust surveillance mechanisms aided with the adoption of telehealth. Effective surveillance on the continent has already been achieved in Nigeria and South Africa making use of mobile network positioning data and application. In Nigeria, partnerships with mobile operators to develop contact tracing and surveillance application of known contacts through mobile positioning data significantly improved decision making, capacity and scope of contact tracing, and surveillance. We noted that collaborations between the government, mobile network operators, and technology companies were utilised for successful mobile positioning data tracing interventions for COVID-19 patients. Mobile applications such as the Vula platform in South Africa were also noted as effective in providing psychological support to health care workers attending to patients [14]. While hotspots areas are quarantined, health care delivery will be continued through telehealth. Africa needs to rapidly adopt and scale-up telehealth. There is an urgent need for enabling policies that standardise telehealth platforms for use based on evidence and best practices with the requisite regulatory oversight. Long-term, telehealth should become mainstreamed into the public health system. Implementing telehealth in the present requires leveraging on the already developed platforms such as Google and Facebook to do contact tracing and identify COVID-19 hotspots [15]. Granted that a lot of education on use and a change management strategy are a prerequisite for telehealth success, governments´ collaboration with health and civic organisations will significantly improve successful outcomes. A recent review of telehealth on the African continent established that smart mobile applications are effective in providing information for referrals of suspected COVID-19 patients and provide convenient access to routine care without the risk of exposure through close contact [14].

**c) Adoption of alternative education platforms:** education is a human right and the COVID-19 pandemic is threatening this right. Schools where applicable must be encouraged to adopt the use of digital technologies for education and promote home-schooling for the delivery of lessons. International support for digital education is forthcoming on different platforms including United Nations Educational, Scientific and Cultural Organization WhatsApp platform and Dzidzo Paden|Imfundwe´ndlini App for remote teaching [16]. Also, several educational technologies such as learning management systems, virtual reality, video-conferencing tools, and mass media communication can be utilised to ensure inclusive education and equitable quality for all where equity, excellence, and students´ well-being are prioritised to reduce learning disparities especially during and post-pandemic [17,18]. Where there is limited internet connectivity home-schooling through postal services and radio broadcasts can be considered as an alternative. The combined effect of these measures increases the capacity for children to continue learning during the period of quarantine or isolation. This pandemic will not be the last and governments need to future proof similar pandemic through investment in schools´ infrastructure to permit implementation of social distancing and infection control measures [19].

**d) COVID-19 vaccine passports:** the vaccine rollout at the end of 2020 to fight the pandemic has provided a glimmer of hope to eliminate the virus. However, Africa and most low to middle-income countries are significantly constrained with access to and uptake of the vaccines [20]. The few countries that have started vaccination on the continent are mulling introducing COVID-19 vaccine passports linked to the identity of the holder. The purpose of vaccination certificates or immunity passports, governments argue, is to allow people to open the economy and return to normal life. Vaccine passports are not a novel idea in Africa these already exist for infections like yellow fever and these have the endorsement of WHO and are recognised under international health regulations. Some argue that vaccine passports provide an incentive for individuals to get vaccinated and thus hasten the pace of achieving herd immunity. Additionally, the vaccine passports will allow for the opening of economic space and mitigate against social, economic, and mental harm caused by lockdowns. There are ethical considerations for COVID-19 passports that individual governments must take into account when developing policy [21], but in the interim, visitors to a host nation should produce a vaccine passport as a mitigatory measure against the importation of COVID-19 variants into the country. Although WHO does not currently endorse COVID-19 vaccination or immunity passports, it has, however, initiated a Smart Vaccination Certificate Working Group to establish key specifications and standards for effective and interoperable digital solutions for COVID-19 vaccination [22]. Emerging technologies such as blockchain, 5 technology, quick response code, Internet of Medical Things could facilitate the development of robust and privacy-preserving COVID-19 vaccination certificates and immunity passports [23]. Some countries have started developing digital applications that generate vaccination certificates and immunity passports. However, currently, there is no international standardised framework or policy guiding the development and implementation of digital tools that generate vaccination certificates and immunity passports [24]. Therefore, for safe easing of COVID-19 restrictions, African countries through Africa Centres for Disease Control and Prevention should consider these digital technologies when developing digital solutions for effective tracing and monitoring emerging variants and infections; rolling out of vaccines, regional sharing, and validation of vaccination certificates and immunity passports for domestic use and international travel, as part of post-pandemic recovery and exit strategies.

## Conclusion

Many governments responded to the COVID-19 pandemic by imposing lockdown and restrictive measures to curb the spread of the disease. Consequently, there was remarkable disruption of ongoing public health programmes, social and economic activities. In light of the negative impact that lockdowns have had on the African continent, we recommend a shift in policy from the imposition of hard lockdowns to smart and targeted lockdowns on COVID-19 hotspots and allow normalcy in less-affected regions. There is an urgent need for the scale-up digital-based models of learning and service delivery to enable continuity of learning.
